# Optic nerve compression associated with visual cortex functional alteration in dysthyroid optic neuropathy: A combined orbital and brain imaging study

**DOI:** 10.1111/cns.14820

**Published:** 2024-07-01

**Authors:** Haiyang Zhang, Yuting Liu, Mengda Jiang, Duojin Xia, Yuhang Peng, Ling Zhu, Xiaofeng Tao, Jue Wang, Jipeng Li, Jing Sun, Yinwei Li, Xuefei Song, Huifang Zhou, Xianqun Fan

**Affiliations:** ^1^ Department of Ophthalmology, Shanghai Ninth People's Hospital Shanghai Jiao Tong University School of Medicine Shanghai China; ^2^ Shanghai Key Laboratory of Orbital Diseases and Ocular Oncology Shanghai China; ^3^ Department of Radiology, Shanghai Ninth People's Hospital Shanghai Jiao Tong University School of Medicine Shanghai China; ^4^ School of Health Science and Engineering University of Shanghai for Science and Technology Shanghai China; ^5^ Institute of Sports Medicine and Health Chengdu Sport University Chengdu China

**Keywords:** dysthyroid optic neuropathy, functional magnetic resonance imaging, optic nerve, vision disorders, visual cortex

## Abstract

**Aims:**

To investigate the alterations of the optic nerve and visual cortex in dysthyroid optic neuropathy (DON), a subgroup of thyroid eye disease (TED).

**Methods:**

Multiple orbital imaging biomarkers related to optic nerve compression and the amplitude of low‐frequency fluctuations (ALFF) of the brain were obtained from 47 patients with DON, 56 TED patients without DON (nDON), and 37 healthy controls (HC). Correlation analyses and diagnostic tests were implemented.

**Results:**

Compared with HC, the nDON group showed alterations in orbital imaging biomarkers related to optic nerve compression in posterior segments, as well as ALFF of the right inferior temporal gyrus and left fusiform gyrus. DON differed from nDON group mainly in the modified muscle index of the posterior segment of optic nerve, and ALFF of orbital part of right superior frontal gyrus, right hippocampus, and right superior temporal gyrus. Orbital and brain imaging biomarkers were significantly correlated with each other. Diagnostic models attained an area under a curve of 0.80 for the detection of DON.

**Conclusion:**

The combined orbital and brain imaging study revealed alterations of the visual pathway in patients with TED and DON as well as provided diagnostic value. The initiation of alterations in the visual cortex in TED may precede the onset of DON.

## Graphical abstract

Changes of in the visual cortex were observed before the progression to dysthyroid optic neuropathy (DON). With the onset of DON, significant intraorbital optic nerve compression was noted, accompanied by further alterations in the visual cortex and other brain regions. This provides novel explanations of visual pathway alterations in DON.

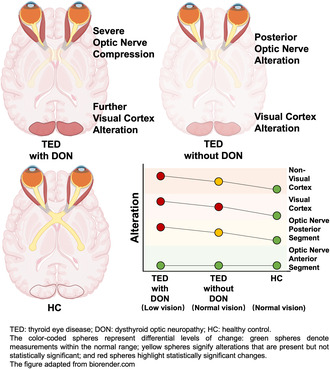

## 1 INTRODUCTION

Thyroid eye disease (TED) is the most common inflammatory orbital disease that poses significant physical and psychological problems, including proptosis, eyeball movement dysfunction, strabismus, exacerbating pain, a decrease in visual acuity, anxiety, depression, etc.^1^, ^2^, ^3^, ^4^, ^5^ Among these problems, visual dysfunction stands out as a paramount concern, leading to a profound decline in patients' quality of life.^6^, ^7^ In most severe conditions, the complication called dysthyroid optic neuropathy (DON) may take place, which decreases visual function of the patients and even deteriorate into blindness.^8^ Early detection and management of visual dysfunction is crucial to preserve the visual health of patients with TED.^9^ However, the precise pathogenesis of DON is not well understood. The primary theoretical basis of DON refers to optic nerve compression (ONC) on account of enlarged and inflamed extraocular muscles (EOMs), especially in the apex of the orbit.^8^, ^10^, ^11^, ^12^, ^13^ Some other risk factors might also be attributable, including abnormal immune modulation, thyroid hormone metabolism, and other undiscovered neurological disorders.^10^ Therefore, more research is needed to unveil the pathogenesis of DON and enhance its precise identification and intervention strategies.

Imaging studies are important for the investigation of orbital alterations in the context of TED and DON. Notably, orbital magnetic resonance imaging (MRI) holds a crucial role.^14^ Barrett et al.^15^ proved that the modified muscle index (mMI) serves as a potent indicator of ONC and diagnostic biomarker of DON. Previous research has also studied other orbital imaging parameters such as optic nerve diameter (OND) and optic nerve sheath diameter (ONSD).^16^ In addition to conventional orbital MRI metrics, novel brain imaging techniques are utilized in TED and DON. Resting‐state functional MRI (rs‐fMRI) is a promising noninvasive functional imaging technique to measure spontaneous brain activities and was applied in TED recently. This has expanded our understanding of DON, revealing its aberrant neurological alterations not only in the intraorbital optic nerve (ON) but also in the visual cortex. Specifically, alterations were found in vision‐related brain regions, like the fusiform gyrus, superior occipital gyrus, and superior temporal gyrus, in patients with DON compared to healthy controls (HCs).^17^, ^18^, ^19^, ^20^, ^21^ This further underscores the complex neurological pathogenesis of DON since the conventional theory of orbital decompression cannot fully explain the visual dysfunction of patients with DON.^22^

To date, few studies have investigated the causative factors of altered visual cortex in TED with or without DON. Given the integrity of the visual pathway, we postulate that there might be associations between ONC, as the mainstream risk factor, and alterations of the visual cortex in the patients. Specifically, we employed orbital MRI to measure the alteration in the anterior and posterior segments of ON. Moreover, we used the amplitude of low‐frequency fluctuation (ALFF) based on rs‐fMRI to detect potential aberrant brain changes. This approach was chosen because ALFF is underpinned by a well‐defined neural mechanism and reflects local neural activity rather than temporal correlations.^23^, ^24^ Later, we conducted correlation analyses of the orbital and brain imaging parameters to delve into the potential influencing factors of the visual cortex changes in TED with or without DON. Therefore, we have devised a combined orbital and brain imaging study to investigate the visual pathway alterations in patients with TED with or without DON, aiming to provide deeper insights into the pathogenesis of optic neuropathy in TED.

## 2 MATERIALS AND METHODS

### 2.1 Participants

This prospective study has received approval from the Ethics Committee of Shanghai Ninth People's Hospital, Shanghai Jiao Tong University School of Medicine (approval number: SH9H‐2022‐T229‐2). A total of 103 patients with TED from Shanghai Ninth People's Hospital, including 47 DON (unilateral, *n* = 15; bilateral, *n* = 32) and 56 nDON (TED patients without DON), along with 36 HC matched in sex, age, and education level, were enrolled in our study from December 2022 to May 2023. Disease duration was determined from the onset of ocular manifestations.

**FIGURE 1:**
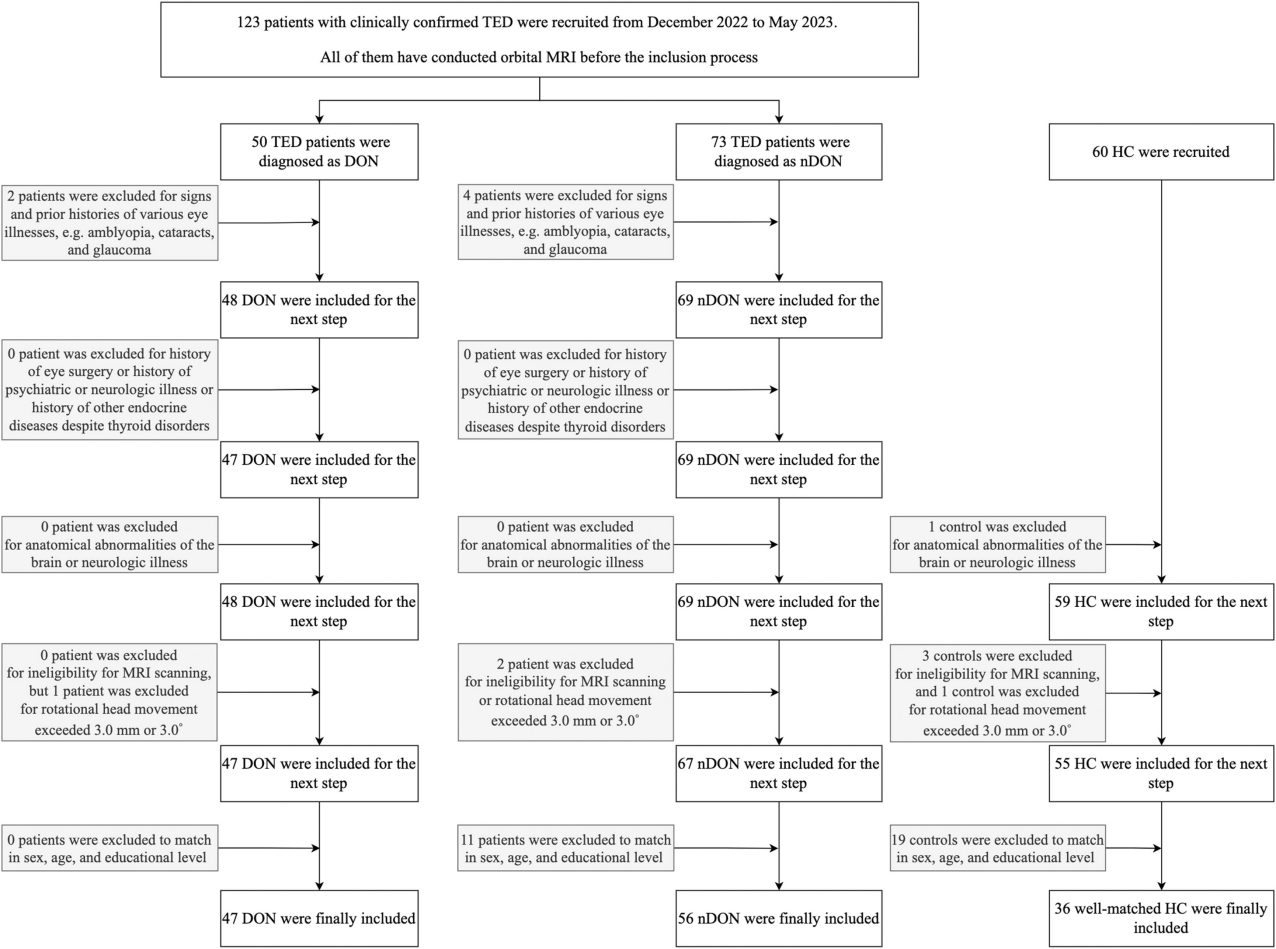
Flowchart of the inclusion process of our study.

For TED, we evaluated several clinical and laboratory characteristics. Clinical characteristics include disease duration, the seven‐point clinical activity score (CAS), best‐corrected visual acuity (BCVA), intraocular pressure (IOP), exophthalmos, and palpebral fissure height. Laboratory characteristics contain thyroid‐stimulating hormone (TSH), free triiodothyronine (fT3), free thyroxine (fT4), and the thyroid‐stimulating hormone receptor antibody (TRAb). In addition, a Graves' orbitopathy‐specific QoL questionnaire (GO‐QoL) including visual functioning and appearance part was conducted.^25^ For HC, BCVA was also examined. The data from the more severely affected eye of patients with DON was included in the analysis, while for the other two groups, the average of the data from both eyes was included in the analysis. Detailed information and exclusion criteria on patients and HC are displayed in Figure 1. The detailed diagnostic standard is shown in Supplementary Material S1.

### 2.2 Imaging data acquisition

A combined orbital and brain imaging study was implemented on each participant. MRI examination was done using a 3.0 Tesla scanner (Magnetom Vida, Siemens, Erlangen, Germany) equipped with a 64‐channel phased array head coil. Head motion and scanning noise were reduced by using foam padding and earplugs. All subjects were required to close their eyes without falling asleep when undergoing MRI scanning. For orbital imaging, Dixon T2‐weighted imaging was applied. For brain imaging, the high‐resolution sagittal structural T1‐weighted images (3D‐T1WI) and functional images covering the whole brain were obtained. Details of sequence parameters are shown in Supplementary Material S2.

### 2.3 Orbital MRI processing

The measurement of orbital imaging parameters associated with intraorbital ON was independently conducted by two orbital radiologists with more than 10 years of experience (operator 1 with 12 years of experience in head and neck radiology; operator 2 with 10 years of experience in head and neck radiology), blinded to clinical information. A comprehensive review of the coronal MRI data of all participants revealed that the ON predominantly resided between retrobulbar layer 0 and retrobulbar layer 6. Due to the presence of vitreous in layer 0 and the difficulties in accurately measuring layer 6 at the apex, the first and fifth retrobulbar layers were chosen for their clearer representation of the anterior (‐A) and posterior (‐P) statues of ON, respectively. All the measurements were performed on the Siemens postprocessing workstation.

**FIGURE 2:**
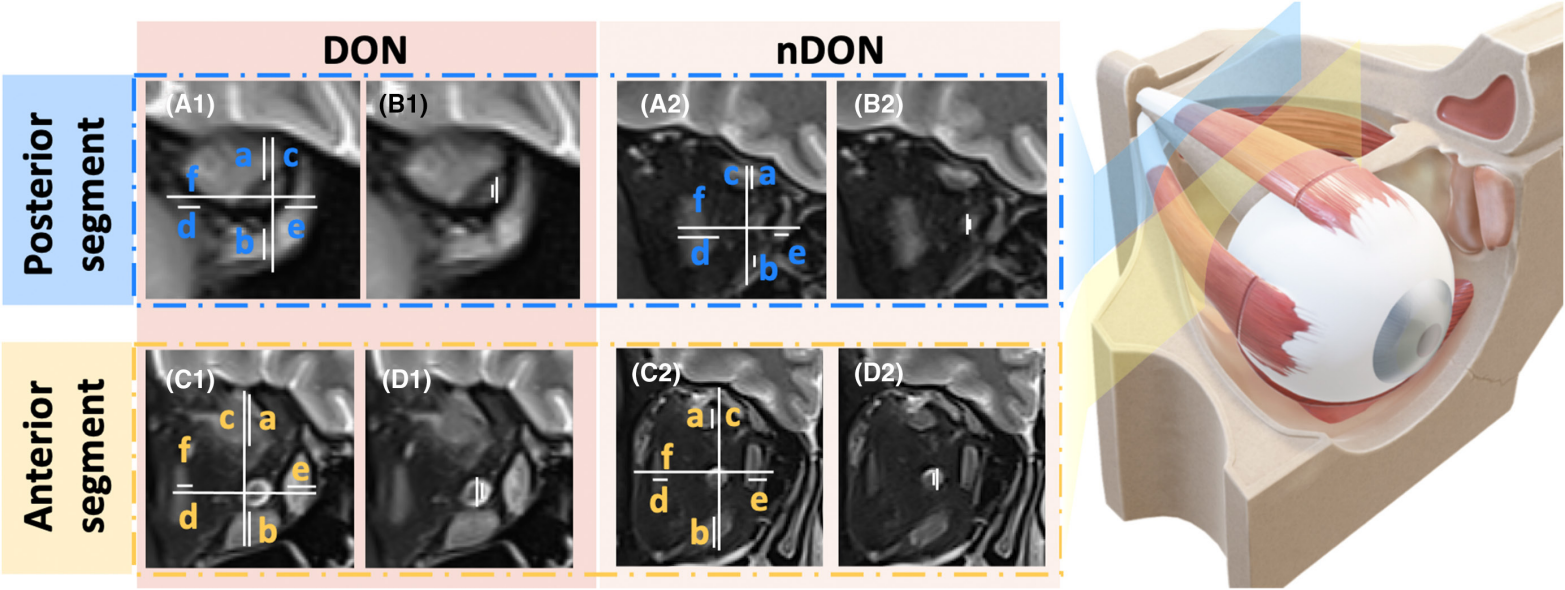
Measurement of orbital imaging parameters associated with intraorbital optic nerve. The yellow and blue planes correspond to the first and fifth retrobulbar layers. The dark red background denotes images of a 65‐year‐old patient with DON marked with “1,” while the light red background signifies images of a 52‐year‐old patient with TED but without DON marked with “2.” (A1 and A2) The image shows the measurement of the modified muscle index on the water map of the Dixon sequence. (B1 and B2) The image shows the measurement of the meridian parameters of the optic nerve sheath and the optic nerve on the fat map of the Dixon sequence.

All measured graphs were reviewed by another orbital radiology expert with 20 years of experience. The coronal Dixon water images were used to measure the ON‐related diameters and mMI. For the diameters, we calculated optic nerve sheath diameter (ONSD) and optic nerve diameter (OND).^26^ For the mMI, it was calculated as the maximum percentage of the orbital width occupied by the extraocular rectus muscles (the maximum value between (*a* + *b*)/*c* and (*d* + *e*)/*f*).^16^ Details are displayed in Figure 2. The intraclass correlation coefficient (ICC) was employed to evaluate the consistency of measurements between the two operators and within each operator individually. An ICC value exceeding the 80% threshold indicates that the variability between operators does not significantly impact the measurement outcomes. Consequently, the average of the measurement values obtained by the two operators was used as the basis for subsequent statistical analyses (Table S1).

### 2.4 Brain rs‐fMRI processing

For imaging data processing, DPABI version 7.0 (http://rfmri.org/DPABI) was used to preprocess all of the rs‐fMRI data based on the MATLAB platform (www.mathworks.com/products/matlab/).^25^ For the purposes of magnetization balancing, the first 10 functional volumes were eliminated. Slice timing and realignment for head motion correction were performed. Images were normalized to the Montreal Neurological Institute EPI template (resampling voxel size = 3 × 3 × 3 mm^3^). A 6‐mm full‐width at half‐maximum Gaussian kernel was applied for smoothing. Detrending was applied to remove linear trends. Finally, the nuisance covariates were eliminated using linear regression, including the average signals from the cerebrospinal fluid and white matter as well as the six head motion parameters.^27^

The amplitude of low‐frequency fluctuation (ALFF) representing the intrinsic neural activity was computed across the whole brain. The time courses were converted to the frequency domain with a fast Fourier transform algorithm to enable each voxel to contain the amplitude of the signal across the whole spectrum. The averaged square root of the spectrum spanning the frequency range of 0.01–0.08 Hz was taken as the ALFF measurement. For standardized variability among the participants, the mean ALFF was obtained as the ALFF value divided by the global mean ALFF value.

### 2.5 Statistical analyses

The demographic and clinical data were analyzed using GraphPad Prism 9 (GraphPad, CA, USA). We assessed normality using the Kolmogorov–Smirnov test. Group differences among DON, nDON, and HC were analyzed as follows: For continuous variables with a Gaussian distribution, one‐way ANOVA was used for comparisons among three groups, and independent‐sample *t*‐tests were employed for two‐group comparisons. For non‐Gaussian distributed continuous data, Kruskal‐Wallis tests were applied for three‐group comparisons, and Mann–Whitney *U* tests were used for two‐group comparisons. Categorical variables were analyzed using chi‐square tests. The level of *p* < 0.05 indicated statistical significance.

The DPABI toolbox 7.0 was used for analyzing ALFF between the three groups.^25^ One‐way ANOVA was conducted to compare the group differences in the ALFF values among the three groups. The post hoc two‐sample *t* tests were conducted to compare the values in brain regions with significant differences between each pair of groups. Multiple comparison correction was conducted within the whole brain, and significant clusters were identified using a false discovery rate (FDR) correction with a two‐tailed test. BrainNet Viewer was used to visualize alterations of brain models.^28^

The relationships between the orbital imaging biomarkers, brain imaging biomarkers, and clinical characteristics related to visual dysfunction were assessed using linear regression models. The threshold for statistical two‐tailed significance was set at *p* < 0.05. Finally, receiver operating characteristic (ROC) curve analysis was conducted to identify DON group from TED without DON group with parameters obtained from orbital MRI and rs‐fMRI, and DeLong's test was employed to compare the performance of different models.

## 3 RESULTS

### 3.1 Demographic and clinical characteristics

Key demographic and clinical characteristics are presented in Table 1. Across all three groups, no significant difference was observed in terms of sex, age, and years of education. A noteworthy distinction was evident in BCVA. Within the two patient groups, DON patients displayed significantly lower BCVA and visual function score of GO‐QoL scale, and exhibited higher levels of TRAb.

**TABLE 1:** Demographic and clinical characteristics of patients with TED and HC.

Characteristics	DON (*n* = 47)	nDON (*n* = 56)	HC (*n* = 36)	*p*‐value			
				DON vs. nDON	DON vs. HC	nDON vs. HC	DON vs. nDON vs. HC
Demographic and TED‐related information							
Sex (male/female)	18/29	22/34	16/20	0.92	0.65	0.67	0.84
Age (year)	50.36 ± 12.26	50.48 ± 9.095	45.64 ± 14.43	0.35	0.11	0.06	0.95
Education (year)	10.43 (9.00, 15.00)	12.00 (9.00, 15.00)	12.00 (9.00, 15.00)	0.06	0.18	0.72	0.15
Smoking (yes/no)	12/35	13/43	/	0.80	/	/	/
Disease duration (month)	14.33 (6.00, 20.00)	17.46 (6.00, 15.00)	/	0.33	/	/	/
CAS	3.23 ± 1.88	2.34 ± 1.56	/	0.95	/	/	/
BCVA	0.34 ± 0.23	0.95 ± 0.08	0.96 ± 0.07	**<0.0001**	**<0.0001**	0.62	**<0.0001**
IOP (mmHg)	20.00 (17.10, 21.00)	18.50 (17.00, 20.50)	/	0.05	/	/	/
Exophthalmos (mm)	19.55 ± 2.88	19.33 ± 2.58	/	0.69	/	/	/
Palpebral fissure height (mm)	9.25 (8.00, 11.00)	10.00 (9.00, 11.00)	/	0.05	/	/	/
Laboratory test information							
TSH (μIU/mL)	0.69 (0.01, 3.27)	1.60 (0.60, 2.54)	/	0.17	/	/	/
fT3 (pg/mL)	3.77 (2.95, 4.89)	3.55 (3.10, 4.26)	/	0.37	/	/	/
fT4 (ng/dL)	1.80 (0.88, 11.20)	1.03 (0.79, 13.94)	/	0.47	/	/	/
TRAb (IU/L)	11.70 (2.68, 16.60)	4.67 (1.10, 15.58)	/	**0.03**	/	/	/
Scale score of GO‐QoL							
Visual functioning	42.50 ± 30.05	54.33 ± 28.57	/	**0.04**	/	/	/
Appearance	58.65 ± 28.48	61.02 ± 33.26	/	0.70	/	/	/

*Note*: Continuous variables are presented as the mean (± standard deviation) for variables with a Gaussian distribution or as the median (interquartile range) for variables with a non‐Gaussian distribution. Bold numbers indicate statistically significant results.

Categorical variables are presented as the number (%) and counts.

### 3.2 Orbital imaging biomarkers related to ONC

**FIGURE 3:**
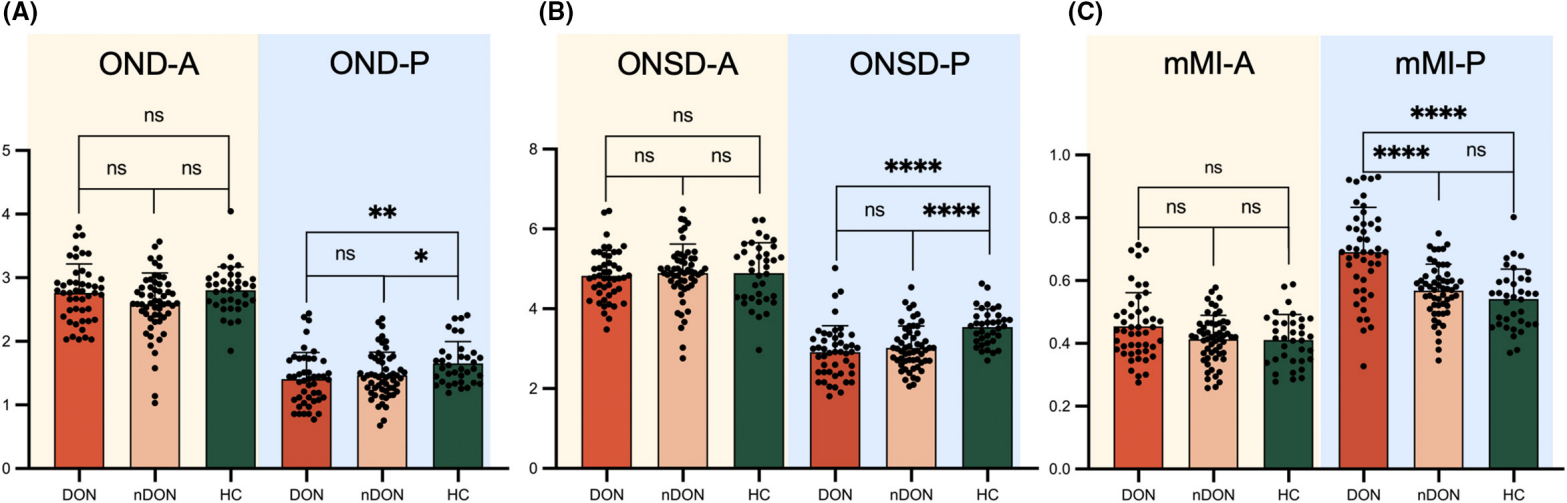
Comparisons of orbital imaging biomarkers among the three groups. ns *p* > = 0.05; *p < 0.05; **p < 0.01; ***p < 0.001; ****p < 0.0001.

Figure 3 summarizes various orbital imaging biomarkers for both the anterior and posterior segments of ON in DON, nDON, and HC groups. Significantly differences in OND‐A, OND‐P, ONSD‐P, and mMI‐P were observed among the three groups. Furthermore, OND‐P and ONSD‐P proved to distinguish between DON and HC, as well as between nDON and HC. However, the only significant difference between the DON and nDON groups was observed in the parameter mMI‐P, as the other parameters did not exhibit the same distinguishing function. Details are demonstrated in Table 2.

**TABLE 2:** Comparison of orbital imaging biomarkers of patients with TED and HC.

Parameters	DON (*n* = 47)	nDON (*n* = 56)	HC (*n* = 36)	*p‐*value			
				DON vs. nDON	DON vs. HC	nDON vs. HC	DON vs. nDON vs. HC
OND‐A	2.80 (2.32, 2.98)	2.62 (2.33, 2.92)	2.82 (2.59, 2.99)	0.06	0.51	**0.02**	**0.04**
ONSD‐A	4.83 (4.39, 5.33)	4.79 (4.52, 5.31)	4.48 (4.17, 5.38)	0.74	0.92	0.52	0.89
mMI‐A	0.45 ± 0.11	0.41 ± 0.08	0.41 ± 0.08	0.36	0.22	0.39	0.29
OND‐P	1.35 (1.05, 1.73)	1.38 (1.20, 1.76)	1.53 (1.35, 1.78)	0.41	**0.00**	**0.01**	**0.02**
ONSD‐P	2.89 (2.38, 3.32)	2.93 (2.67, 3.40)	3.29 (3.13, 3.82)	0.28	**<0.0001**	**<0.0001**	**<0.0001**
mMI‐P	0.69 ± 0.15	0.57 ± 0.10	0.55 ± 0.09	**<0.0001**	**<0.0001**	0.14	**<0.0001**

*Note*: Continuous variables are presented as the mean (± standard deviation) for variables with a Gaussian distribution or as the median (interquartile range) for variables with a non‐Gaussian distribution.

The notations ‘A’ and ‘P’ appended to the indicator correspond to the first and fifth retrobulbar layers on the coronal MRI, which shows the anterior and posterior parts of the optic nerve.

Bold numbers indicate statistically significant results.

### 3.3 Imaging biomarkers of ALFF

**FIGURE 4:**
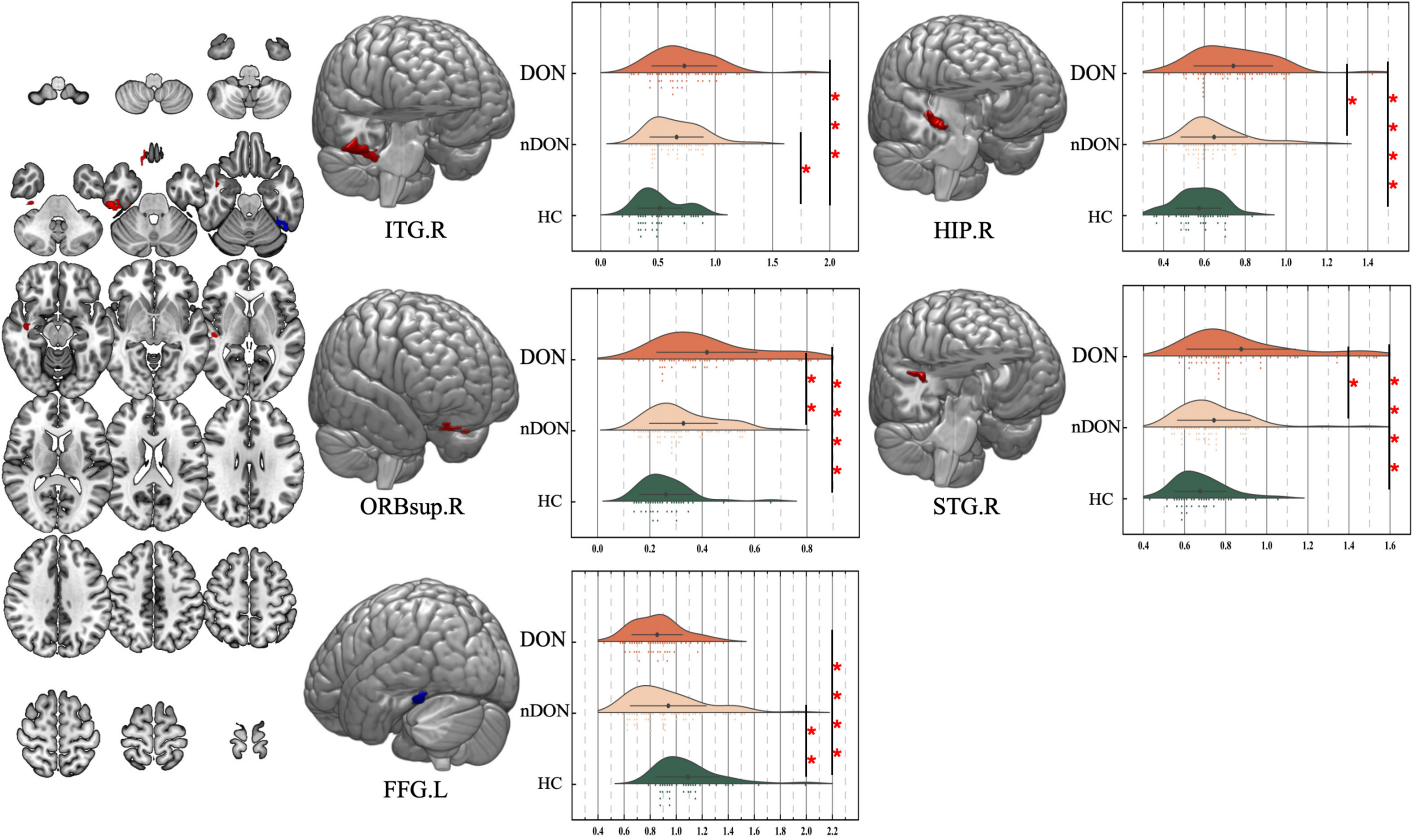
Comparisons of the altered amplitude of low‐frequency fluctuations during the resting state among the three groups. The differences primarily existed in the FFG.L, ORBsup.R, HIP.R, STG.R, and ITG.R (*p‐*corr < 0.05, FDR corrected). *p < 0.05; **p < 0.01; ***p < 0.001; ****p < 0.0001.

The results of the one‐way ANOVA analysis showed that the ALFF was significantly different in the left fusiform gyrus (FFG.L), right superior frontal gyrus, orbital part (ORBsup.R), right hippocampus (HIP.R), right superior temporal gyrus (STG.R), and right inferior temporal gyrus (ITG.R). The brain regions distinguishing nDON from HC included ITG.R and FFG.L. In contrast, the distinctive regions between DON and nDON encompassed ORBsup.R, HIP.R, and STG.R. (Figure 4; Table 3).

**TABLE 3:** Abnormal amplitude of low‐frequency fluctuation in patients with TED.

Conditions	Brain regions	Brodmann area	Cluster size	MNI coordinates of peak voxel			*F* value
				*X*	*Y*	*Z*	
DON < HC, nDON < HC							
	Left fusiform gyrus	37	20	−45	−52	−23	11.374
DON > HC, DON > nDON							
	Right superior frontal gyrus, orbital part	11, 47	14	15	33	−30	11.830
	Right hippocampus	54	33	39	−11	−15	13.204
	Right superior temporal gyrus	41	20	45	−21	3	10.865
DON > HC, nDON > HC							
	Right inferior temporal gyrus	20	70	54	−24	−28	11.882

*Note*: False discovery rate correction, *p*‐corr = 0.05.

### 3.4 Correlation analyses of ONC and visual cortex alteration

**FIGURE 5:**
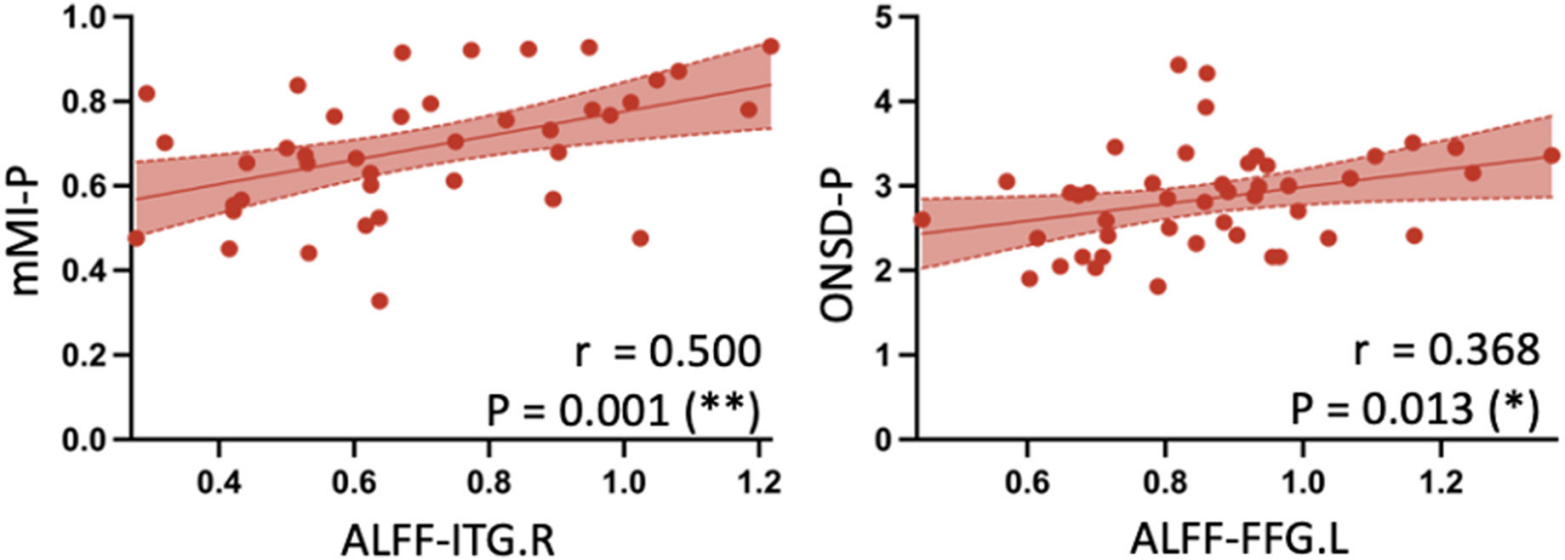
Correlation analysis between imaging biomarkers related to ONC and visual cortex alteration in the patients with DON. Dark lines depict linear regression with a 95% confidence interval (shadow in red).

In the DON group, significant correlations emerged: ALFF‐ITG.R correlated with mMI‐P (*r* = 0.500, *p* = 0.001). Moreover, ALFF‐FFG.L showed significant correlations with ONSD‐P (*r* = 0.368, *p* = 0.013) (Figure 5). Additional correlation details are shown in Figure S1.

### 3.5 Clinical relevance and diagnostic model construction

**FIGURE 6:**
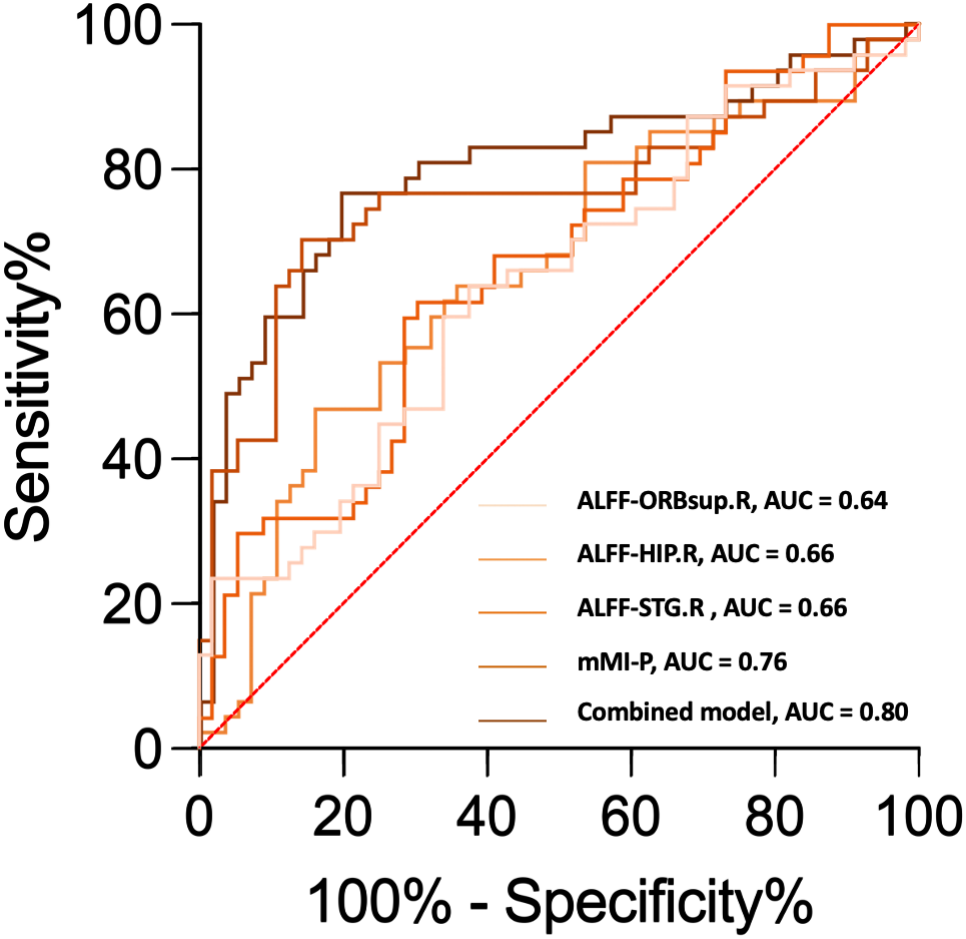
The ROC curve for identification of DON within the TED population with orbital and brain imaging biomarkers.

Discriminative orbital imaging biomarkers and brain imaging biomarkers were integrated into the univariate analysis. In the aspect of diagnosing DON, ROC analysis indicated that the structural parameter, mMI‐P, exhibited superior diagnostic capability compared to the imaging biomarkers ALFF‐ORBsup.R, ALFF‐HIP.R, and ALFF‐STG.R, with AUC values of 0.76, 0.64, 0.66, and 0.66, respectively. The mMI‐P and ALFF values of HIP.R and STG.R were enrolled in the multivariate logistic regression analysis. The combined model demonstrated enhanced diagnostic performance with an AUC of 0.80 (Table 4; Figure 6). However, further analysis using Delong's test showed that the best performing univariate model (mMI‐P) and the multivariate model did not have a significant difference in diagnostic performance (*p* = 0.1456).

**TABLE 4:** Diagnostic performance of orbital and brain imaging biomarkers for identification of DON.

Parameter	DON vs. nDON		
	AUC	95% confidence interval	*p*‐value
Univariate analysis			
ALFF‐ORBsup.R	0.64	(0.53, 0.75)	**0.02**
ALFF‐HIP.R	0.66	(0.55, 0.77)	**0.01**
ALFF‐STG.R	0.66	(0.56, 0.77)	**0.00**
mMI‐P	0.76	(0.66, 0.86)	**0.00**
Multivariate analysis			
Combined model	0.80	(0.71, 0.89)	**0.00**

*Note*: Bold numbers indicate statistically significant results.

Combined model: model combining ALFF of HIP.R, STG.R, and mMI‐P.

## 4 DISCUSSION

The alteration of the entire visual pathway in DON remains a pivotal, yet unresolved issue in pathogenesis research. The relationship between the primary theoretical basis, ONC, and the recently discovered visual cortex alterations needs to be elucidated. MRI emerges as a crucial tool for addressing this challenge. Specifically, rs‐fMRI stands out as a method to investigate the functional changes of the brain.^29^ As the DON rs‐fMRI study with maximum subject sample size so far, our study identified significant alterations mainly in posterior segment of intraorbital ON, while not in anterior segment. In addition, we observed changes in brain activity within specific brain regions, namely the ORBsup.R, HIP.R, and STG.R in patients with DON compared to nDON. In instances of TED patients without DON, alterations manifested in the ITG.R and FFG.L compared to HC, which are integral parts of the visual cortex. Our combined orbital and brain imaging study provides fresh insights into the elusive pathogenesis of DON.

The prevailing theory of ONC attributes the primary mechanism to the orbital fibroblast deposition of hyaluronic acid, causing enlargement of EOMs, orbital fat, and increased vascular congestion. This culminates in significant compression of the intraorbital ON, ultimately leading to ON ischemia or the hindrance of axonal flow.^10^ By measuring OND, ONSD, and mMI at two levels behind the eyeball, we explored the ONC status of the anterior and posterior segments. The results revealed that only biomarkers from the posterior segment of ON held the discriminatory capacity between TED with or without DON and HC. No significant change was observed in the anterior segment of ON in any group. Compared to HC, TED patients without DON exhibited significantly decreased posterior OND and ONSD, rather than mMI. This observation suggests that in patients with TED who have not yet progressed to DON, intraorbital ON might have already undergone significant structural changes. These changes might be attributed to other reasons such as the increased volume of orbital fat, rather than the enlargement of EOMs highlighted in clinical situations.^30^ However, among the three orbital imaging biomarkers related to ONC we investigated, only the posterior mMI exhibited statistical significance when comparing TED patients with or without DON. Since the posterior mMI reflects the proportion of EOMs in the orbit, intuitively indicating compression in the orbital apex, our findings align with and supplement the primary mechanism of DON.^10^ In general, when considering TED patients with or without DON, it becomes apparent that imaging biomarkers obtained from the posterior segment of the ON exert a pronounced influence on the progression of the disease. We noticed that a prior research on normal‐pressure glaucoma also holds the same opinion that MRI biomarkers obtained at the orbital apex have higher correlation coefficients.^31^ This suggests the significance of orbital imaging parameters at the orbital apex in understanding and studying ON disorders. Hence, future research should focus on optimizing imaging parameter acquisition at the orbital apex.

Apart from the most studied DON mechanism as ONC, it is crucial to delve into alterations in the brain, especially the visual cortex, and understand their contribution to the pathogenesis. Observable differences were noted in both FFG.L and ITG.R between TED patients without DON and HC, which are integral components of the dorsal visual pathway in the visual cortex, primarily responsible for target and object recognition.^32^ Previous investigations examining TED patients without DON have also identified distinct changes in the same regions in multiple aspects including increased connectivity, reduced regional neurovascular coupling, etc.^19^, ^33^, ^34^, ^35^, ^36^ Therefore, we postulate that before the onset of DON, TED patients might have already undergone inflammatory changes throughout the entire visual pathway, encompassing the ON and the visual cortex. Despite the fact that the mMI in TED patients without DON exhibited minimal changes, and that the BCVA that is primarily determined by the status of ON also remained largely unaltered, structural modifications (OND and ONSD) occurred in the intraorbital ON due to ONC. These alterations, coupled with changes in the visual cortex, may contribute to the decline in the visual functioning score in TED patients without DON in comparison to HC.^37^, ^38^ Additional ophthalmic assessments of visual function are essential to elucidate the specific mechanisms underlying its impact on vision.

When comparing TED patients with or without DON, alterations in the ORBsup.R, HIP.R, and STG.R were observed. These brain regions play a prominent role in functions associated with cognitive decision‐making, memory, auditory, and language processing, not directly associated with visual processing.^39^, ^40^, ^41^ Although there is common speculation that DON patients should have more pronounced changes in the entire visual pathway compared to TED patients without DON, both our results and previous imaging studies have not shown significant alterations in the visual cortex within the DON group.^17^, ^19^, ^42^, ^43^ Our observation may suggest that the primary cause of DON is more likely associated with severe apex ONC derived from enlargement of EOMs (posterior mMI). This peripheral compression mechanism, rather than the subsequent visual cortex alterations, severely damages the intraorbital ON and significantly impaired BCVA and visual function. However, it should be acknowledged that in the progression of TED without DON to DON occurrence, the brain regions of various function did further alter and deserve further investigations.

Through correlation analyses in both TED with and without DON groups, positive interplays were unveiled between alterations in the visual cortex and the orbital ONC. In DON group, activity in ITG.R positively correlated with posterior mMI, and activity in FFG.L positively correlated with posterior ONSD. Considering only the posterior mMI could distinguish DON and nDON, we speculated that the alterations in the visual cortex in DON might be secondary to ONC and evolve as ONC progresses. A future longitudinal study would offer more robust evidence to support this speculative mechanism. In addition, the orbital imaging biomarkers and brain imaging biomarkers identified in our study demonstrated commendable diagnostic potential of DON. The combined model using posterior mMI and ALFF value of three brain regions exhibited superior identification ability compared to single‐biomarker models, attaining an AUC of 0.80. Given the challenging nature of diagnosing DON in clinical practice, particularly when elderly individuals present confounding factors, such as comorbid ophthalmic diseases that influence clinical and electrophysiological examinations, the use of objective neuroimaging indicators may offer more reliable evidence for the identification of DON.^44^

There are some limitations of our study. Firstly, even though our study represents the largest subject sample size in TED neuroimaging to date, we recognize that the sample size is modest when compared to neuroimaging studies in broader fields. A larger sample size would help us draw more stable and reliable conclusion. Secondly, as a cross‐sectional study, we were unable to reveal an absolute causal relationship between intraorbital ON and visual cortex in TED with or without DON. In future research, we intend to investigate the neuropathological mechanisms of DON and TED by employing animal models and conducting histopathological examination. Thirdly, this study utilizing rs‐fMRI and orbital Dixon imaging is not yet comprehensive in explaining the changes in the entire visual pathway. In subsequent research, we aspire to employ more multimodal MRI technologies (eg. diffusion tensor imaging, T2‐weighted‐Fluid‐Attenuated Inversion Recovery sequence, etc.) to explore the functional changes of the ON, optic tract, lateral geniculate body, and other relevant parts of the visual pathway, as well as incorporate advanced imaging processing and analytical methods (e.g. graph theory and brain network analysis). Finally, our current investigation relied on a limited evaluation of visual function using the BCVA and GO‐QoL scale. To enhance the depth of assessment, future studies will incorporate more ophthalmic examinations and questionnaires regarding different aspects of visual function.

## 5 CONCLUSIONS

Our combined orbital and brain imaging research has revealed distinct alterations in the posterior segment of intraorbital ON and various brain regions in both TED patients with or without DON. The observed changes in visual cortex were closely associated with the presence of ONC. Our findings underscore the importance of directing attention to the alteration of the entire visual pathway in TED patients even before the onset of DON. Correspondingly, orbital and brain imaging markers are of great significance for precise identification of DON. Our research contributes to a deeper understanding of the pathogenesis underlying visual pathway alteration and visual dysfunction in DON and TED patients without DON. In the future, more in‐depth mechanistic exploration studies are needed to shed light on such important and meaningful phenomenon.

## CONFLICT OF INTEREST STATEMENT

The authors declare that they have no known competing financial interests or personal relationships that could have appeared to influence the work reported in this article.

## Supporting information


Data S1

## Data Availability

The data that support the findings of this study are available from the corresponding author upon reasonable request.
