# Supplementary material for: Optic nerve compression associated with visual cortex functional alteration in dysthyroid optic neuropathy: A combined orbital and brain imaging study
Source: CNS Neurosci Ther. 2024 Jul 1;30(7):e14820. doi: 10.1111/cns.14820 (PMC11215484; doi:10.1111/cns.14820)
Supplement: Supplementary file 1 — Data S1 [file CNS-30-e14820-s001.docx]

**Optic Nerve Compression Associated with Visual Cortex Functional Alteration in Dysthyroid Optic Neuropathy: A Combined Orbital and Brain Imaging Study**

**Supplementary Material 1**: The diagnostic standard.

TED was diagnosed based on the clinical guidelines proposed by the European Group on Graves' Orbitopathy (EUGOGO).^1^

DON was determined by a prerequisite history of TED and at least two of the following subsequent manifestations: (1) decreased best-corrected visual acuity (BCVA) < 0.8; (2) abnormal pattern-visual evoked potentials (delayed and/or amplitude reduction); (3) papilledema; (4) visual field defects; and (5) abnormal color vision .^2^

**Supplementary material 2**: Details of MRI sequences.

For orbital imaging, Dixon T2 weighted imaging was applied with the following parameters: repetition time [TR] = 3800 ms; echo time [TE] = 94 ms; number of slices =15; thickness = 3 mm; matrix = 256 × 256 mm^2^; field of view [FOV]= 180 × 180 mm^2^; and flip angle [FA]= 160°.

For brain imaging, the high-resolution sagittal structural T1-weighted images (3D-T1WI) with the following parameters (TR = 2,400 ms; TE = 2.4 ms; thickness = 0.8 mm; acquisition matrix = 320 × 320 mm^2^; FOV = 256 × 256 mm^2^; FA = 8°; number of slices = 208; voxel size = 0.8 mm isotropic) and functional images with the parameters (TR = 2000 ms; TE = 30 ms; thickness = 2.0 mm; acquisition matrix = 104 ×104 mm^2^; FOV = 208 × 208 mm^2^; FA = 90°; number of slices = 72; voxel size = 2mm isotropic) covering the whole brain were obtained. The total duration of the 3D-T1WI was 6 minutes and 52 seconds, and for the rs-fMRI, it was 8 minutes and 13 seconds.

**Supplementary Figure 1**

**
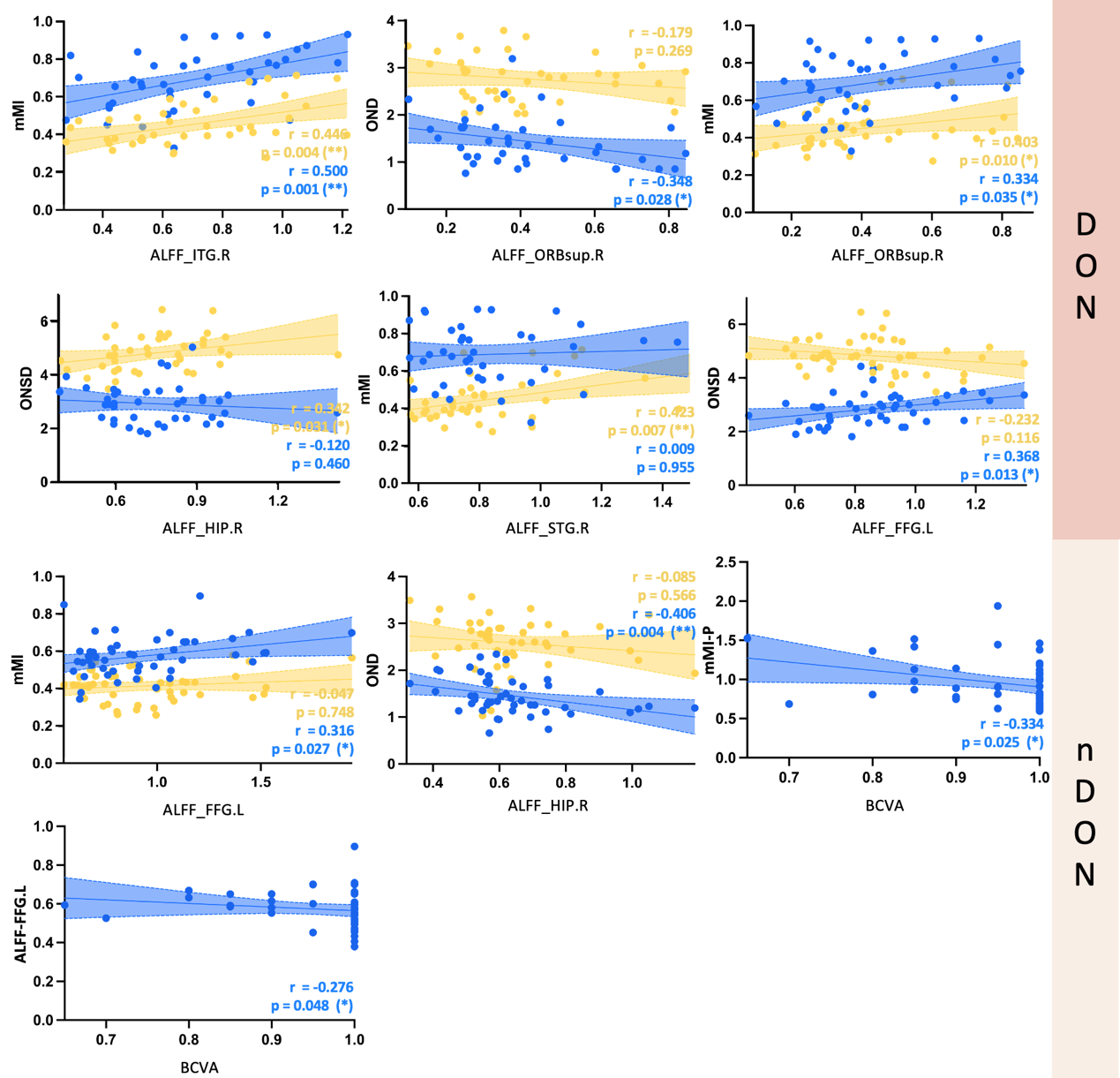
**

Correlation analysis between the orbital imaging biomarkers, brain imaging biomarkers, and clinical characteristics related to visual dysfunction in TED patients with or without DON. Dark lines depict linear regression with a 95% confidence interval (shadow in blue or yellow). The yellow curve corresponds to orbital imaging biomarkers obtained from the anterior ON segment, while the blue curve corresponds to biomarkers from the posterior ON segment.

**Supplementary Table 1**

Inter-operator and intra-operator ICCs for measurements of multiple parameters with two different operators.

| Parameters | ICC (95%CI) | |
| --- | --- | --- |
|  | Inter-operator | Intra-operator |
| OND-A | 0.973 (0.862-0.986) | 0.969 (0.846-0.973) |
| ONSD-A | 0.965 (0.802-0.983) | 0.935 (0.842-0.986) |
| mMI-A | 0.888 (0.830-0.931) | 0.876 (0.825-0.912) |
| OND-P | 0.884 (0.786-0.964) | 0.939 (0.886-0.973) |
| ONSD-P | 0.871 (0.855-0.869) | 0.874 (0.859-0.891) |
| mMI-P | 0.913 (0.856-0.923) | 0.856 (0.808-0.893) |
| The notations 'A' and 'P' appended to the indicator correspond to locations 3 mm and 15 mm posterior to the eyeball on the MRI, which shows the anterior and posterior parts of the optic nerve. | | |

**References：**

1. Bartalena L, Kahaly GJ, Baldeschi L, et al. The 2021 European Group on Graves’ orbitopathy (EUGOGO) clinical practice guidelines for the medical management of Graves’ orbitopathy. *European Journal of Endocrinology*. 2021;185(4):G43-G67. doi:10.1530/EJE-21-0479

2. Song C, Luo Y, Huang W, et al. Extraocular muscle volume index at the orbital apex with optic neuritis: a combined parameter for diagnosis of dysthyroid optic neuropathy. *Eur Radiol*. Published online July 5, 2023. doi:10.1007/s00330-023-09848-x
